# P154: Sanitize the vehicle

**DOI:** 10.1186/2047-2994-2-S1-P154

**Published:** 2013-06-20

**Authors:** S Dudziak, E Bradshaw, L Joseph-Massiah, J Clark

**Affiliations:** 1 Revera Inc, Mississauga, Canada; 2 Revera Inc, Toronto, Canada; 3 Revera Inc, Ridgetown, Canada

## Introduction

In 2011 driven by our growing concern regarding delivery of care related to infection control and the transmission of infections a hand hygiene project was initiated.

## Objectives

Increase product accessibility at point of care

Improved knowledge of hand hygiene and overall infection control processes

Increase in hand hygiene compliance

## Methods

A review of the following items was completed using the LEAN methodology, the 5 "Why”.

- **System Change** -Reviewed location of alcohol-based hand rub at point of care, reviewed accessibility of alcohol-based hand rub to Patients in wheelchair, reviewed accessibility to water soap and towels, reviewed accessibility to hand moisturizers, Interdisciplinary team involvement.

- **Training / Education** -Family, Patients, staff, visitors, and outside contractors were trained on the moments of hand hygiene and correct procedures for hand rubbing and hand washing, Trained staff to complete hand hygiene observation audits, Staff reviews utilization of gloves, All staff watched a hand hygiene video.

- **Evaluation and feedback** -Assessed staffs perception of hand hygiene – thru focused groups and surveys, Hand hygiene focused observation audits were completed prior to initiating the project and post implementation, Completed environmental Infection control audits to supplement infection control processes overall.

- **Reminders in the workplace** -Poster were posted in public areas, audit results were posted in the quality board for all to see.

- **Institutional safety climate** -Nurtured a culture of Patients safety, Implemented Patients hand hygiene champions on each floor, Involved Patients and Family council.

## Results

- Baseline hand observation audits were compiled and repeated every 3 months. Increase in hand hygiene from 67% to 96% (increase of 29%)

- No outbreaks in 2011, 2012

## Conclusion

This project showed a benefit for the Patients, staff and the community at large.

## Disclosure of interest

None declared
